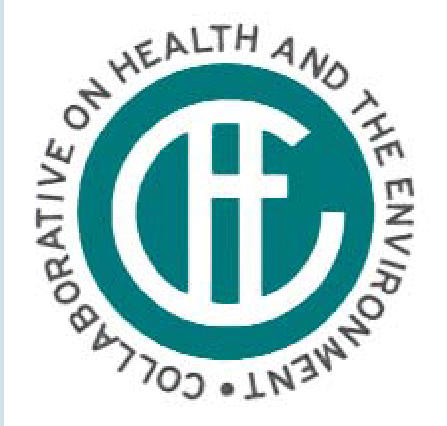# EHPnet: Collaborative on Health and the Environment Toxicant and Disease Database

**Published:** 2006-09

**Authors:** Erin E. Dooley

From huge industrial spills to exposure to everyday products, there are a number of ways people come in contact with potentially hazardous chemicals. To help educate those wanting more in-depth information about the health effects of chemicals, the Collaborative on Health and the Environment (CHE) has developed the CHE Toxicant and Disease Database, available online at **http://database.healthandenvironment.org/**.

The CHE was formed in 2002 as a project of the nonprofit health and environmental research institute Commonweal with a mission to foster a greater understanding of the links between human health and the environment. The database, which summarizes links between chemical contaminants and approximately 180 human diseases and conditions, has been enhanced recently with new search features and a directory of links to other resources that would be of interest to visitors to the CHE site.

Background information on the theory behind the database project is available through a link in the text paragraph near the top of the homepage. Here, visitors can learn about the nature of gene–environment interactions and their impacts on human health; the lack of toxicity data on many of the 80,000-plus chemicals that have been developed, distributed, and discarded over the past 50 years; and the difficulties encountered in assessing the risk of chemical exposures. This page also describes where the information in the database came from, how chemicals are categorized, and what limitations there are to the database.

The homepage features an alphabetical listing of diseases and conditions—ranging from abnormal sperm to Wilm’s tumor—that can potentially be triggered by exposure to environmental chemicals. Each disease or condition links to a page listing its chemically related causes. These causes are grouped by the strength of evidence—strong, good, or limited—of the relationship between the health effect and the chemical. Each page also cites references. Choosing one of the listed chemical causes brings up a record of all of the diseases and conditions linked to that chemical.

The database can be searched using one of the pull-down lists at the right of the homepage. There are options for searching by any of 25 disease categories, by individual disease, or by any of dozens of toxicants. Searches can also be performed by Chemical Abstract Service number and by keyword.

The directory available through the Links to Other Databases and Resources link cites 17 databases, including ones hosted by the NIEHS, the CDC, the EPA, the ATSDR, and the International Agency for Research on Cancer. Other resources listed include the CDC’s *National Report on Human Exposure to Environmental Chemicals*, the National Toxicology Program and its *11th Report on Carcinogens*, the California Office of Environmental Health Hazard Assessment, and *EHP*.

## Figures and Tables

**Figure:**